# Transcriptional suppression of microRNA-27a contributes to laryngeal cancer differentiation via GSK-3β-involved Wnt/β-catenin pathway

**DOI:** 10.18632/oncotarget.14769

**Published:** 2017-01-20

**Authors:** Sheng Chen, Yuan-Yuan Sun, Zhao-Xiong Zhang, Yun-Hui Li, Zhen-Ming Xu, Wei-Neng Fu

**Affiliations:** ^1^ Department of Medical Genetics, China Medical University, Shenyang, 110122, P.R. China; ^2^ Department of Laboratory Medicine, No. 202 Hospital of PLA, Shenyang, 110003, P.R. China; ^3^ Department of Otolaryngology, No. 463 Hospital of PLA, Shenyang, 110007, P.R. China

**Keywords:** laryngeal cancer, miR-27a, ATRA, differentiation, GSK-3β

## Abstract

miR-27a regulates cell differentiation in a variety of diseases. However, whether and how miR-27a participates in laryngeal cancer cell differentiation remains unknown. Therefore, we explored role and molecular mechanism of miR-27a in laryngeal cancer differentiation in the study. We found that miR-27a expression was inversely correlated with laryngeal cancer differentiation degree based on the clinical pathological diagnosis of each patient. miR-27 asignificantly rescued differentiation and inhibited β-catenin, LEF1, OCT4 and SOX2 in Wnt/β-catenin pathway in all-trans-retinoic acid (ATRA)-induced laryngeal cancer cells. Bindings of RARα to miR-27a and miR-27a to GSK-3β were confirmed by ChIP and Luciferase reporter assays, respectively. In conclusion, miR-27a is a negative regulator in laryngeal cancer differentiation. RARα-mediated miR-27a transcriptional inactivation releases the inhibition of miR-27a on GSK-3β leading to laryngeal cancer differentiation through GSK-3β-involved Wnt/β-catenin pathway, suggesting that miR-27a is a usefully therapeutic target at least in ATRA-induced laryngeal cancer differentiation.

## INTRODUCTION

Abnormal differentiation is commonly found in cancer [1, 2]. Studies have shown that cancer is initiated and maintained by a subset of cancer cells, ‘the cancer stem cells’, which are able to self-renew and differentiate like normal stem cells presenting cell surface markers [3–5]. Inducement of cancer cell differentiation has become a promising strategy in modern antineoplastic therapy [6]. Thus, study on molecular mechanism of cancer differentiation is still a hotspot at present.

It is well-known that all-trans-retinoic acid (ATRA) is a powerful agent commonly used in cancer differentiation study [7–10]. Retinoid-involved signal is often disrupted during carcinogenesis and restoration of the signaling may be a viable option for cancer prevention [11]. Retinoid induces cell differentiation through a lot of signal pathways such as PI3K/Akt and Wnt/β-catenin pathways. In retinoid-inducing differentiation, GSK-3β coordinates the crosstalk between the PI3K/Akt and Wnt/β-catenin pathways. ATRA and retinoic acid receptor (RAR) complex inactivate Akt through PI3K/Akt pathway and prevent GSK-3β from phosphorylation by Akt leading to maintenance of GSK-3β activity [12]. GSK-3β then phosphorylates β-catenin and promotes its degradation mediated by ubiquitin in Wnt/β-catenin pathway [13]. As a result, β-catenin cannot enter nuclear to transactivate the differentiation inhibitors such as OCT4 and SOX2 via TCF/LEF [14–16].

In addition, RAR also acts as a transcriptional factor. Studies have shown that RAR regulates transcription of target genes usually together with retinoid X receptor (RXR). For example, GM-CSF/RA-induced RALDH2 transcription in dendritic cells requires binding of cooperative RAR/RXR complex to Sp1 sites of the RALDH2 promoter through p38MAPK-associated pathway [17].

MicroRNA, a 20–23 nt functional RNA molecule, is involved in some biological processes including embryonic development and mammalian cell differentiation [18]. It has been reported that miR-27a is an important differentiation-associated regulator in a variety of diseases [19–21]. However, whether and how miR-27a participates in laryngeal cancer cell differentiation remains unknown.

By prediction, we found that there exists a potential retinoic acid response element (RARE) in the miR-27a promoter whereas GSK-3β is a putative target gene of miR-27a. We wonder miR-27a could regulate differentiation through GSK-3β and RARα-related signaling pathways.

## RESULTS

### miR-27a expression is negatively associated with differentiation degree in laryngeal cancer tissues

**Figure 1:**
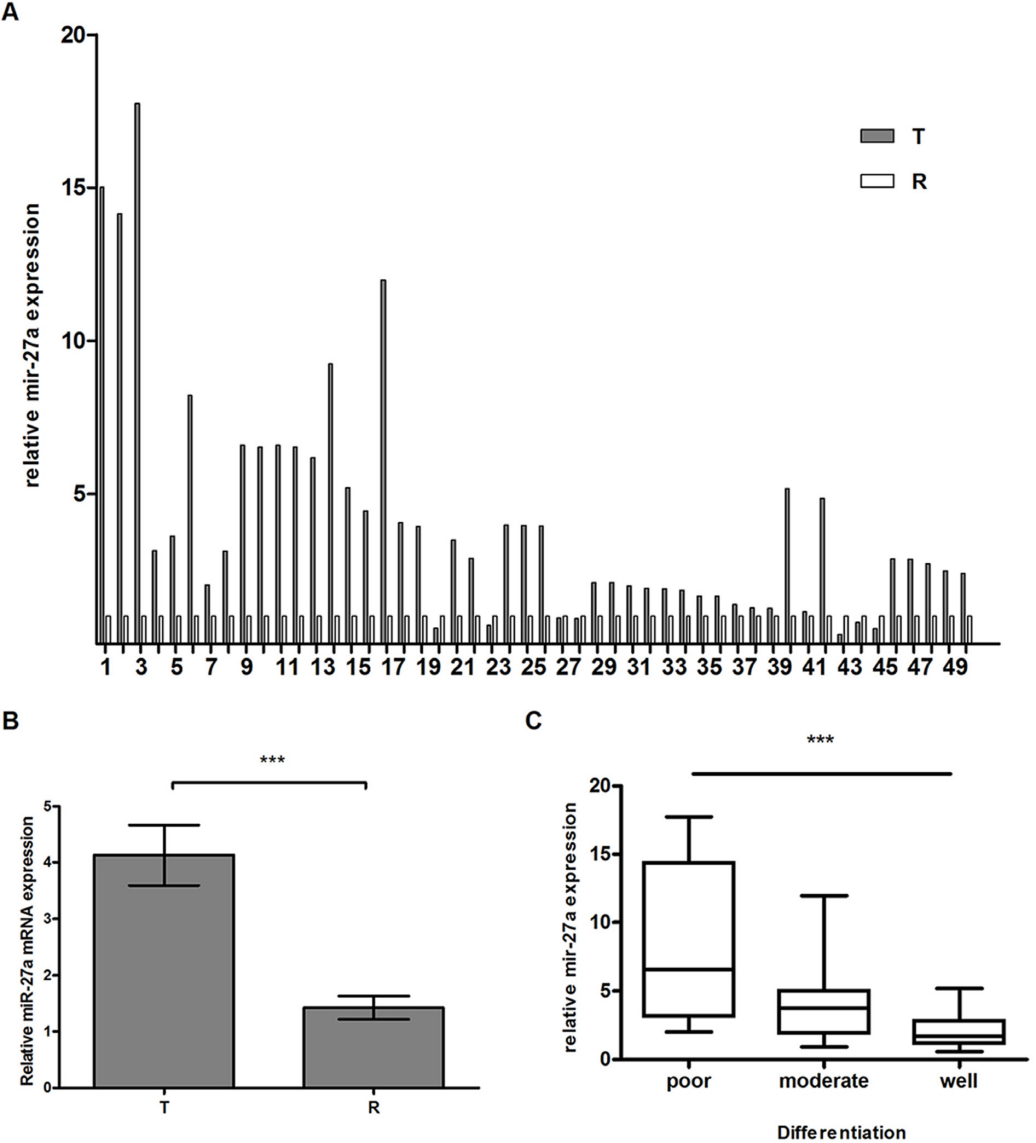
miR-27a expression in laryngeal cancer by RT-qPCR (**A**) miR-27a expression in 50 pairs of LSCC tissues by RT-qPCR. The y-axis indicates relative miR-27a expression in cancer tissues and paired normal adjacent tissues. The relative expression was calculated as the ratio of miR-27a to the internal control, using the equation RQ = 2^–ΔΔCT^ in each sample. The x-axis represents the number of the paired samples used in the study. U6 snRNA was used for the internal control. (**B**) miR27a average expression in LSCC. T and R represent cancer tissues and paired normal adjacent tissues, respectively. (**C**) Box-and-whiskers plot analysis of miR-27a expression in different degrees of differentiated tissues. Data are mean ± SEM of at least three independent experiments. *** indicates *p* < 0.001.

RT-qPCR result demonstrated that miR-27a was up-regulated in 86% (43 of 50) cases of laryngeal cancer (Figure 1A) and miR27a average expression was significantly higher in LSCC tissue (4.131 ± 0.537) than that in adjacent normal tissue (1.4201 ± 0.206) (Figure 1B, *P* < 0.001), suggesting that miR-27a is involved in laryngeal oncogenesis.

As shown in Figure 1C and Table 1, we found that the higher miR-27a level in laryngeal carcinoma was, the lower the differentiation degree was (poor, 8.016 ± 1.790; moderate, 3.843 ± 0.560; well, 2.136 ± 0.336), which showed significant differences between miR-27a expression and differentiation degree (*P* < 0.001), indicating that miR-27a is a negative regulator in laryngeal cancer differentiation.

**Table 1:** Association between miR-27a and clinical characteristics in 50 patients with LSCC

Features	No. of cases	miR-27aexpression (mean ± SEM)	*P*-value
**Age**			
< 60	22	4.964 ± 0.939	0.1607
≥ 60	28	3.431 ± 0.606	
**Gender**			
male	42	4.074 ± 0.581	0.8944
female	8	4.272 ± 1.540	
**Smoking**			
smoker	40	4.100 ± 0.593	0.9835
nonsmoker	10	4.128 ± 1.358	
**Drinking**			
drinker	36	4.340 ± 0.967	0.7895
nondrinker	14	4.014 ± 0.656	
**Differentiation**			
well	16	8.016 ± 1.790	0.0002***
moderate	24	3.843 ± 0.560	
poor	10	2.136 ± 0.336	
**Lymph node**			
negative	31	2.859 ± 0.491	0.0023**
positive	19	6.139 ± 1.030	
**Clinical stage**			
I	7	2.155 ± 0.346	0.0006***
II	15	2.805 ± 0.827	
III	23	4.282 ± 0.689	
IV	5	9.928 ± 2.263	

Notes: One-way ANOVOA was used to analyze the correlation between the miR-27a expression and clinical features of each patient. ** and *** indicates *P* < 0.01 and *P* < 0.001, respectively.

### miR-27a resists ATRA-induced laryngeal cancer cell differentiation

**Figure 2:**
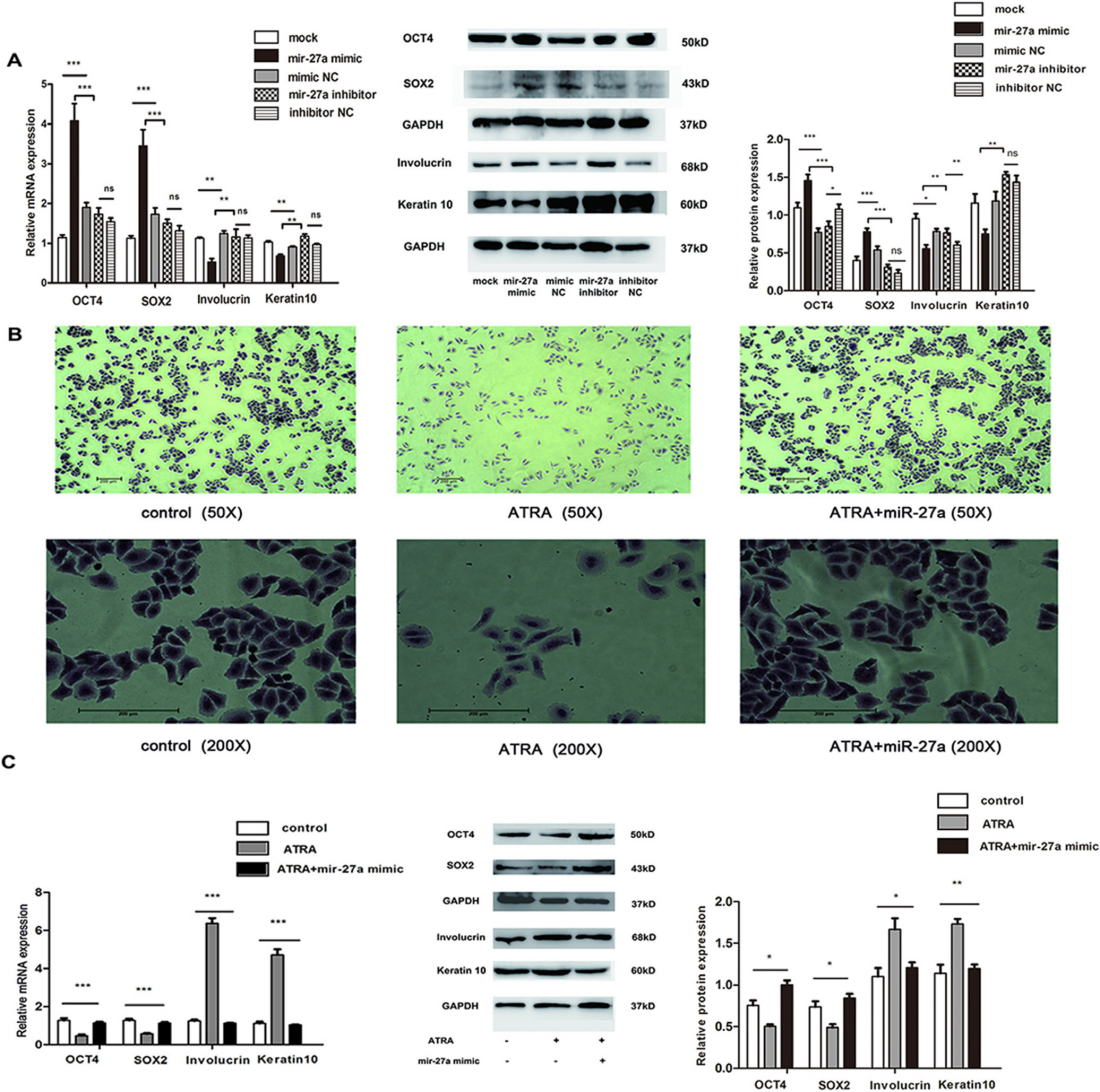
Role of miR-27a in ATRA-induced laryngeal cancer cell differentiation (**A**) Expression of stemness/differentiated markers in Hep2 cells transfected by miR-27a. mRNA and protein levels of each gene were measured by RT-qPCR and Western blot, respectively. GAPDH was used for the internal control. (**B**) Effect of ATRA and miR-27a on Hep-2 cell morphology. (**C**) Effect of miR-27a on expression of stemness/differentiated markers in ATRA-induced Hep2 cells. mRNA and protein levels of each gene were measured by RT-qPCR and Western blot, respectively. GAPDH was used for the internal control. Data were indicated as the mean ± SD from at least three independent experiments. *, ** and *** indicate *p* < 0.05, *p* < 0.01and *p* < 0.001, respectively.

In order to explore whether miR-27a takes part in laryngeal cancer cell differentiation, we first detected effect of miR-27a on expression of stem cell markers and differentiation-associated keratinocyte markers in non-treated Hep2 cells. RT-qPCR and Western blotting results showed that miR-27a mimic significantly increased OCT4 and SOX2 expression both at mRNA and protein levels in Hep2 cells compared to the mock, miR-27a inhibitor and mimic NC, respectively. Conversely, miR-27a mimic significantly reduced Involucrin and Keratin10 expression. However, not all markers showed significant difference between miR-27a inhibitor and inhibitor NC (Figure 2A). These results imply that miR-27a potentially represses laryngeal cancer cell differentiation.

We then explored effect of miR-27a on differentiation in ATRA-induced Hep2 cells. As shown in Supplementary Figure 1, RA concentration at IC50 was approximately 15 μM. Because 5μM RA had relatively low effect on Hep2 cell viability (Supplementary Figure 1) and induced obvious laryngeal cancer cell differentiation (Supplementary Figure 2), 5μM RA was used in the following experiments.

Under light microscope, we then found that non-treated Hep2 cells cultured *in vitro* were shown to have a polygon body with mass nucleocytoplasmic ratio and rapid growth (Figure 2B, left) whereas long shuttle cell bodies, cell marginal shrivel, small nucleocytoplasmic ratio and slow growth were observed in the ATRA-induced Hep2 cells (Figure 2B, middle), which could be blocked by miR-27a introduction (Figure 2B, right). Moreover, significantly lower expression of *OCT4* and *SOX2* and higher expression of *Involucrin* and *Keratin10* at both mRNA and protein levels were found in Hep2 cells treated with 5 μM RA for 48 h compared to the controls, respectively. Nevertheless, the expression alterations of the four markers could be restored by miR-27a (Figure 2C). These results suggest a suppressive role of miR-27a in ATRA-induced laryngeal cancer cell differentiation.

### Mir-27a directly targets *GSK-3β* and increases expression of β-catenin and LEF1 in ATRA-induced Hep2 cells

**Figure 3:**
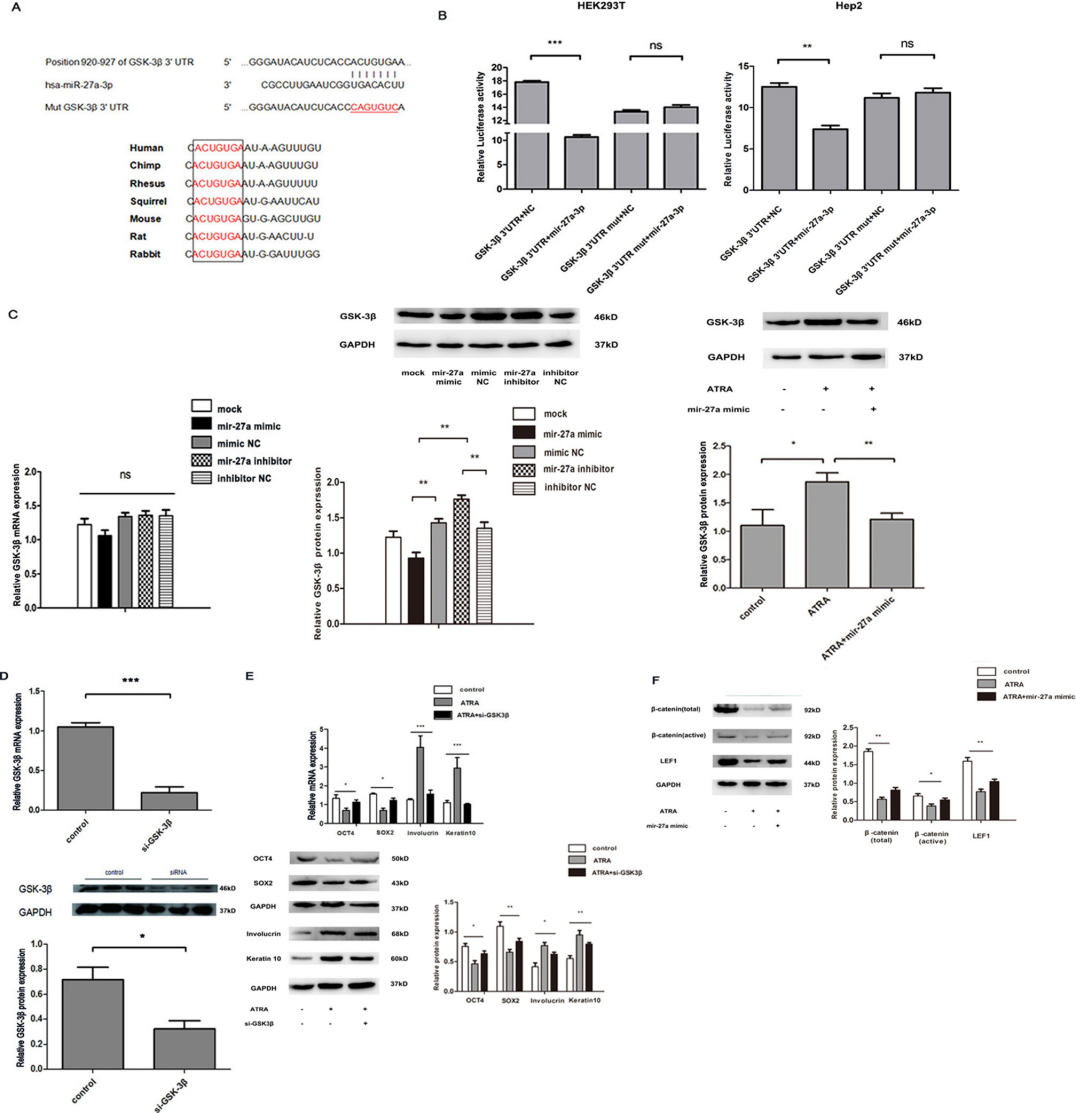
Effects of miR-27a on ATRA-induced Hep2 cells by direct targeting to GSK-3β (**A**) Prediction of miR-27a binding site in the 3′ UTR of GSK-3*β* among vertebrates. (**B**) Binding detection of miR-27a to the *GSK-3β* 3’UTR in HEK293T and Hep2 cells. Luciferase activity of HEK293T and Hep2 cells cotransfected with different constructs was detected. Each value of luciferase activity was calculated as the ratio of firefly to *Renilla*. (**C**) Effect of miR-27a on GSK-3β expression in Hep2 cells. Left, mRNA level in untreated Hep2 cells; Middle, protein level in untreated Hep2 cells; Right, protein level in ATRA-induced Hep2 cells. (**D**) Knockdown of GSK-3β by its interference RNA. (**E**) Effects of GSK-3β knockdown on expression of stemness/differentiated markers in ATRA-induced Hep2 cells. (**F**) Effects of GSK3β knockdown on expression of β-catenin and LEF1 in Wnt/β-catenin signaling pathway in ATRA-induced Hep2 cells. mRNA and protein levels of each gene above were measured by RT-qPCR and Western blot, respectively. GAPDH was used for the internal control. Data were expressed as the mean ± SD from at least three independent experiments. *, **, *** and ns represent *p* < 0.05, *p* < 0.01, *p* < 0.001 and no significance, respectively.

Based on the bioinformatics analysis using different programs, we found a highly-conserved miR-27a targeting sequence in the *GSK-3*β 3′-untranslated region (Figure 3A), which suggests that *GSK-3β* mRNA is a candidate target of miR-27a. Dual luciferase reporter assay result displayed that luciferase activity was significantly reduced in HEK293T and Hep2 cells when *GSK-3β* 3’UTR wild-type construct was cotransfected with miR-27a mimic compared to the controls (Figure 3B). Western blot and real-time RT-qPCR results showed that miR-27a significantly decreased *GSK-3β* expression at protein level but not at mRNA level (Figure 3C, left and middle), respectively, implying that miR-27a represses *GSK-3β* expression from post-transcriptional level. Moreover, ATRA significantly increased the GSK3β gene expression, which was restored by miR-27a (Figure 3C, right). These results indicate that miR-27a directly inhibits *GSK-3β* in ATRA-induced Hep2 cells.

Whether *GSK-3β* is a mediator in miR-27a-regulated differentiation of laryngeal cancer cells induced by ATRA is also a problem. As indicated in Figure 3D, the *GSK-3β* gene was significantly knocked down at both mRNA and protein levels in laryngeal cancer cells transfected with si-*GSK-3β* compared to the control, which means the *GSK-3β* gene is successfully silenced by its small interference RNA. Similar to miR-27a, *GSK-3β* kockdown significantly altered differentiation-associated markers in ATRA-induced Hep2 cells (Figure 3E). These suggest that miR-27a regulates ATRA-induced Hep2 cell differentiation via down-regulating GSK-3β expression.

We also assessed influence of miR-27a in expression of *β-catenin and LEF1* in Wnt/β-catenin signal in ATRA-induced Hep2 cells. We found that ATRA significantly decreased β-catenin and LEF1 expression compared to the controls, respectively, and such effects of ATRA were significantly rescued by miR-27a (Figure 3F), indicating that miR-27a increases β-catenin and LEF1 in ATRA-induced Hep2 cells.

### RARα contributes to transcriptional silence of miR-27a in ATRA-induced laryngeal cancer cell differentiation

**Figure 4:**
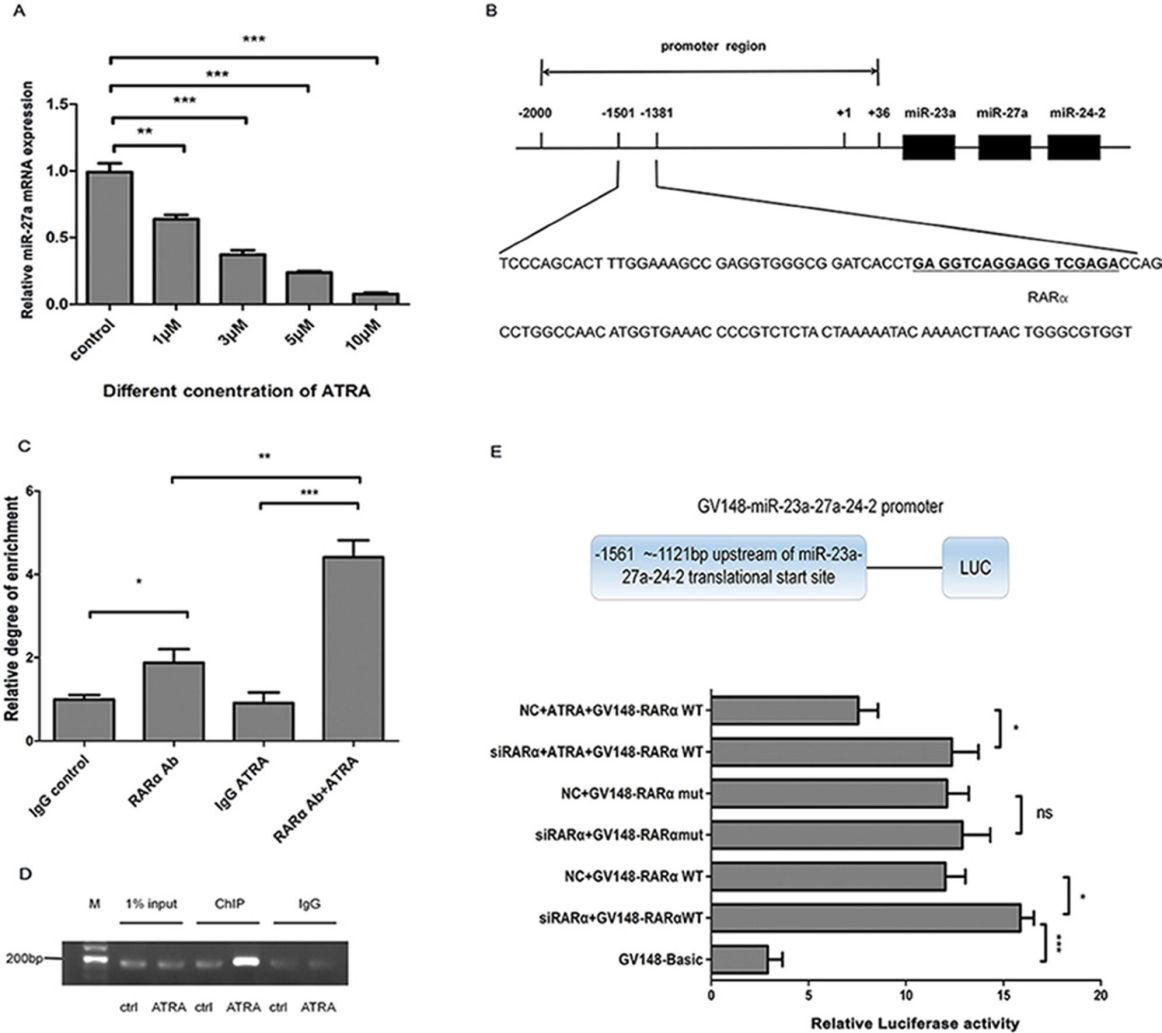
Transcriptional inhibition of miR-27a by RARα in ATRA-induced Hep2 cells (**A**) miR-27a expression in different dosage of ATRA in Hep2 cells. Mature miR-27a was detected using RT-qPCR. U6 snRNA was used for the internal control. (**B**) Predicted RARα binding site within the miR-27a gene promoter region. (**C**) ChIP-qPCR analysis of RARα binding to the miR-27a gene promoter region. (**D**) ChIP-traditional PCR analysis of RARα binding to the miR-27a gene promoter region in ATRA-induced Hep2 cells. DNA immunoprecipitated by anti-RARα antibody from Hep2 cells after treatment with ATRA for 48 h was amplified by PCR to examine abundance of the target sequence. Input DNA and isotope-matched anti-IgG (anti-immunoglobulinG) antibody was used as controls, respectively. (**E**) Effect of RARα on miR-27a transcription activity by Luciferase Reporter assay. GV148-miR-27a wild/mutant promoters, Renilla luciferase plasmids and si-RARα/NC small RNAs were cotransfected into Hep2 cells, respectively. Data were expressed as the mean ± SD from at least three independent experiments. *, **, *** and ns represent *p* < 0.05, *p* < 0.01, *p* < 0.001 and no significance, respectively.

Interestingly, we found that miR-27a level was significantly lower in ATRA-treated Hep2 cells in a dose-dependent manner compared to the controls (Figure 4A). Therefore, how ATRA downregulates miR-27a expression is the other issue we have to solve in the study.

It is well-known that RAR*α* is an important receptor of ATRA. According to bioinformatics prediction, we found a potential RARE site in the *miR-27a* gene promoter region (Figure 4B). By ChIP assay, we obtained a PCR product including RARE site of *miR-27a* promoter from chromatin fragment precipitated by anti-RARα antibody, which confirms the direct binding of RARα to *miR-27a* promoter region. Furthermore, RARα exhibited significantly strong binding to RARE located within the *miR-27a* promoter in ATRA-induced Hep2 cells compared to the controls (Figure 4C and 4D). Luciferase reporter assay result showed that ATRA significantly decreased transcriptional activity of the *miR-27a* promoter containing RARE compared to the controls and si-RARα significantly rescued the effect (Figure 4E), indicating that ATRA/RARα negatively regulate miR-27a transcriptional activity.

Collectively, these results above implied that ATRA promotes RARα binding to *miR-27a* promoter, leading to miR-27a transcription inhibition.

## DISCUSSION

In recent years, miR-27a has been a new star in differentiation research area. Studies have shown that miR-27a is a critical differentiation regulator in various kinds of cells such as myofibroblast [22], embryonic stem cell (ESC) [23], adipocyte [24], osteoblast [25], myoblast [26], erythroid [27] and breast cancer cells [28]. Except for breast cancer, role of miR-27a in other cancer differentiation is seldom reported. In addition, whether miR-27a affects ATAR-induced differentiation is not reported either.

In the study, we found that miR-27a overexpression is negatively associated with differentiation degree in laryngeal cancer tissues and suppresses differentiation of Hep2 cells mediated by ATRA. miR-27a also maintains OCT4 and SOX2 expression and suppresses Involucrin and Keratin10 expression in ATRA-treated and -untreated Hep2 cells. These findings suggest that miR-27a inhibits laryngeal cancer differentiation.

Same to our findings, miR-27a represses differentiation in lung fibroblast [22], ESC [23] and adipocyte [24]. However, miR-27a is also found to promote osteoblast [25], myoblast [26], erythroid [27] and breast cancer stem like cell differentiation [28].These indicates that miR-27a has dual effects on differentiation in different kinds of cells via targeting various of genes through different signal pathways. For examples, miR-27a targets α-smooth muscle actin, Smad2 and Smad4 to inhibit lung fibroblast differentiation [22]. miR-27a prevents adipocyte differentiation through direct bind to Pparγ [24]. miR-27a enhances osteoblast differentiation through targeting sFRP1 in canonical Wnt/β-catenin signaling [25].

Studies have shown that ATRA promotes translocation of phosphorylated p38MAPK into nuclear leading to activation of Msk1 whereas phosphorylated Msk1 recruits transcription factors such as RARα to the promoter region of their targets [29, 30]. In the study, we found that RARα directly binds to the miR-27a gene promoter region and decrease its transcription activity in Hep2 cells. We speculate that RARα directly inhibits miR-27a transcription probably via p38MAPK-involved pathway.

As indicated in the Introduction, ATRA/RAR*α* maintains GSK-3β activity though PI3K/Akt pathway and promotes differentiation via GSK-3β-involved Wnt/β-catenin pathway. In Wnt/β-catenin pathway, GSK-3β blocks translocation of β-catenin to nuclear to activate differentiation inhibitors such as OCT4 and SOX2 through LEF1 leading to differentiation.

In the study, we found that miR-27a itself directly binds to GSK-3β 3′-UTR in both HEK293T and Hep2 cells and inhibits the gene expression in Hep2 cells. Interestingly, ATRA also decreased the expression of miR-27a.Whereas in ATRA-treated Hep2 cells, the RARα, GSK-3β, Involucrin and Keratin10 genes are upregulated and β-catenin, LEF1, OCT4 and SOX2 genes are downregulated. Moreover, the expression alterations of these genes can be reversed by miR-27a in ATRA-induced Hep2 cells, indicating that miR-27a blocks ATRA-induced Hep2 cell differentiation through GSK-3β-mediated Wnt/β-catenin pathway by direct target to GSK-3β.

**Figure 5:**
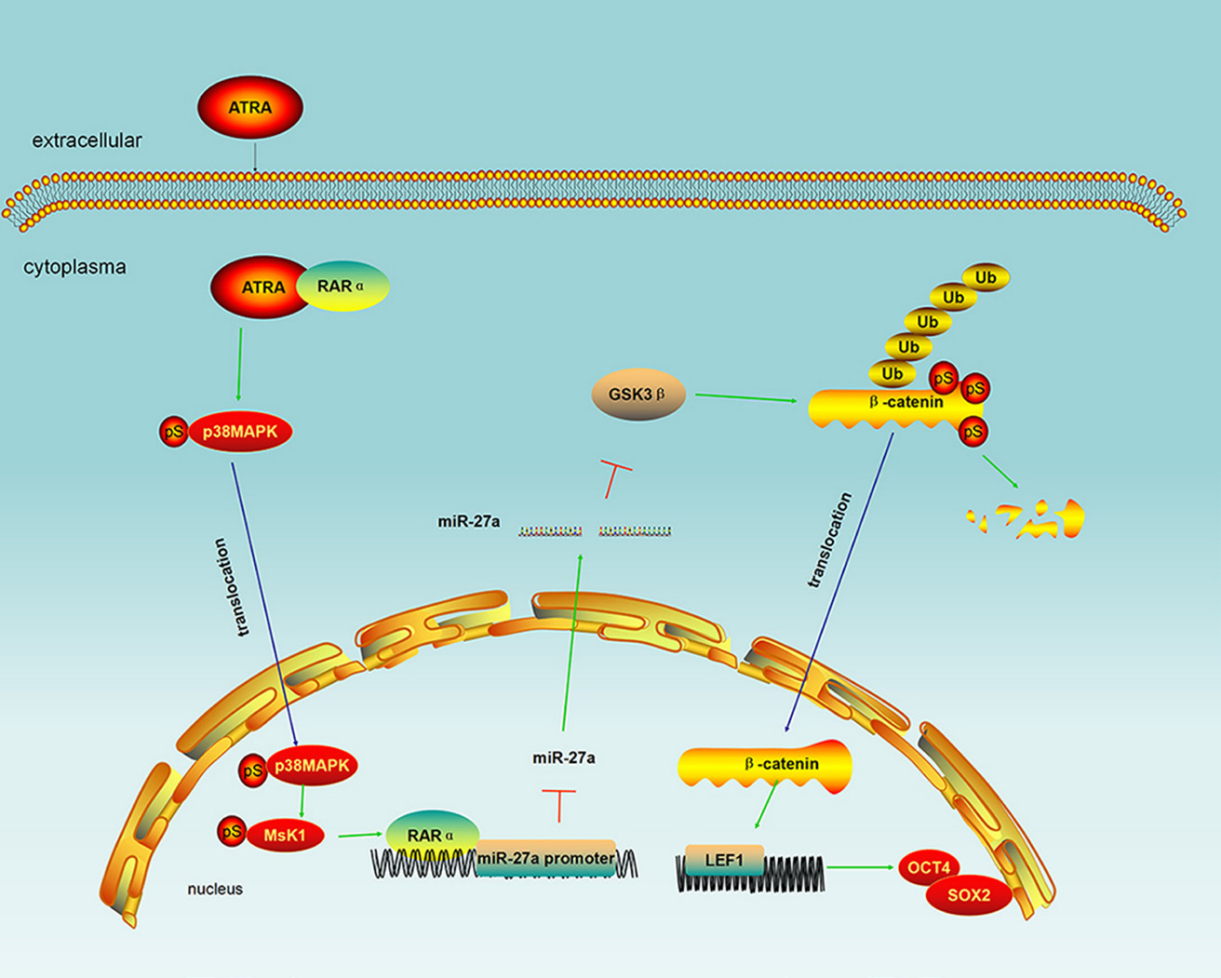
Schematic illustration of miR-27a in ATRA/RARα and GSK-3β-involved Wnt/β-catenin pathways

In conclusion, miR-27a plays a suppressive role in laryngeal cancer differentiation. ATRA enhances the suppressive role of RARα on miR-27a promoter and release the inhibition of miR-27a on GSK-3β leading to laryngeal cancer differentiation through GSK-3β-involved Wnt/β-catenin pathway (Figure 5). We believe that miR-27a is a usefully therapeutic target at least in ATRA-induced laryngeal cancer differentiation.

## MATERIALS AND METHODS

### Patient tissues and cell lines

Tissue specimens including tumor tissues and matched non-tumor tissues from 50 laryngeal squamous cell carcinoma (LSCC) patients were collected after they gave informed consent. Verification of the specimens was performed by a pathologist and the samples were immediately frozen at −80°C after been removed from the patients. Human laryngeal cancer cells Hep2 and human embryonic kidneycells HEK293T were obtained from the KeyGEN BioTECH Company of Jiangsu Province and were maintained in RPMI 1640 (GIBCO, LA, CA) with 10% new-born calf serum (Hyclone, Logan, USA) and DMEM (GIBCO, LA, CA) with 10% fetal bovine serum (Hyclone, Logan, USA), 100 units/ml penicillin and 100μg/ml streptomycin in a humidified atmosphere at 37°C in 5% CO_2_.

### Gene transfection

Cell-based experiments were carried out by transfection of 20nM miRNA duplex (GenePharma, Shanghai, China), non-relative control RNA duplex (NC duplex, GenePharma) and small interfering RNA (siRNA, GenePharma) into the Hep2 cells using Lipofectamine™ 3000 (Invitrogen, USA) in accordance with the manufacturer's procedure. Sequences of the corresponding small non-coding RNAs were shown in Table 2.

**Table 2:** Nucleotide sequences used in the study

Name	Sequence
miR-27a mimics	5′-UUCACAGUGGCUAAGUUCCGC-3′
miR-27a inhibitor	5′-GCGGAACUUAGCCACUGUGAA-3′
mimic NC	5′-UUCUCCGAACGUGUCACGUTT-3′
inhibitor NC	5′-CAGUACUUUUGUGUAGUACAA-3′
si-GSK3β	5′-GGACAAGAGAUUUAAGAAUTT-3′
si-RARα	5′-GCUUCCAGUUAGUGGAUAUTT-3′
NC	5′-GGCUACGUCCAGGAGCGCACC-3′
MiR-27a(F)	5′- TTCACAGTGGCTAAGTTCCGC-3′
U6 primers(F)	5′-CTCGCTTCGGCAGCACA-3′
U6 primers(R)	5′-AACGCTTCACGAATTTGCGT-3′
Keratin10(F)	5′- TCCCAACTGGCCTTGAAACA-3′
Keratin10(R)	5′- AGGCTGCGGTAGGTTTGAAT-3′
Involucrin(F)	5′- AGCCTAAGEATCTGGAGCAG-3′
Involucrin(R)	5′-AGGGCTGGTTGAATGTCTTG-3′
OCT4(F)	5′-GCAATTTGCCAAGCTCCTGAA-3′
OCT4(R)	5′-GCAGATGGTCGTTTGGCTGA-3′
SOX2(F)	5′- CCTCCAGTCAATACCCATCA-3′
SOX2(R)	5′- TTCTTGCTCAGGCAGTCC-3′
GSK-3β(F)	5′- GACTAAGGTCTTCCGACCCC -3′
GSK-3β(R)	5′- TTGAATCCGAGCATGAGGAGG -3′
ChIP primer(F)	5′-GAGGTCAGGAGGTCGAGACC-3′
ChIP primer(R)	5′-CCAGGCTGGAGTGCAATGG-3′

Notes: NC, negative control; si, small interfering; F, forward primer; R, reverse primer.

### RNA isolation and RT-qPCR

Total RNA was extracted from the specimens and the cells using Trizol (Takara, Dalian, China) according to the manufacturer's instructions. Concentrations of total RNA were measured by reading the absorbance at OD260/280 nm.

RT-qPCR was carried out using the ABI 7500 Real Time PCR system (Applied Biosystems, Foster City, CA, USA). First-strand cDNAs for mRNA and miRNA were obtained using the Reverse Transcription Kit (Takara, Dalian, China) and TransScript miRNA First-Strand cDNA Synthesis SuperMix (TransGen Biotech, Beijing, China) in accordance with the manufacturer's procedure, respectively. Quantitative PCR was performed using SYBR^®^ Premix Ex Taq™ II (Takara) according to the manufacturer's instructions. GAPDH mRNA and endogenous U6 small nuclear RNA (snRNA) levels were assayed for normalization, respectively. 2^-ΔΔCt^ method was used for relative quantification and the primer sequences used in the study were shown in Table 2.

### Western blot

Cells were lysed in RIPA cell lysis buffer (Beyotime, Shanghai, China) in the presence of protease inhibitor cocktail (Biotool, Huston, USA) and PMSF (Beyotime). Protein concentration was quantified by BCA protein assay kit (Beyotime). 50μg of the extracts were separated on 10% SDS-PAGE and transferred to PVDF membrane. Membrane was then blocked with 5% non-fat milk and incubated overnight with the following primary antibodies, respectively, which are Keratin10 (Abcam, Cambridge, USA), Involucrin (Proteintech, Wuhan, China), OCT4 (Proteintech), SOX2 (Proteintech), GSK-3β (Abcam), β-catenin (Proteintech), non-phospho β-catenin (Cell Signaling, Boston, USA) and LEF1 (Proteintech) followed by incubation with appropriate secondary antibodies conjugated to horseradish peroxidase (HRP) for 1 h. Hybridization signal was detected by enhanced chemi-luminescence (ECL) (ThermoFisher, MA,USA) according to the manufacturer's instructions. GAPDH (ZSGB-BIO, Guangdong, China) was used as reference protein and determined following the same procedure as above.

### Cell viability assay

Hep2 cells were transferred to a 96-well plate at a density of 2–3 × 10^3^ cells per well in serum-free culture conditions, treated by different concentrations of ATRA (Sigma–Aldrich, St. Louis, MO, USA) and cultured for 1, 2, 3 or 4 days, respectively. Absorbance at 450 nm was measured using Model 680 Microplate Reader (Bio-Rad, Hercules, CA, USA) after incubation of the cells with 10 μl CCK8 (KeyGEN, Jiangsu, China) for 4 h at 37°C.

### Cell staining and morphology

Hep2 cells were incubated for 48 hours at 37°C in a 5% CO2 incubator in 6-well plates after ATRA treatment. Cells were then fixed with methanol, stained with hematoxylin and eosin, and subjected to light microscopic inspection and photographed.

### Luciferase reporter assay

GV148-GSK3β-3′UTR, GV148-GSK3β-3’UTR-mut, GV272-miR-27a promoter-wild and GV272-miR-27a promoter-mut plasmids were obtained from GENECHEM (Shanghai, China). HEK293T or Hep2 cells seeded in 96-well plate in triplicate were cotransfected with GV148-GSK3β-3′UTR, GV148-GSK3β-3′UTR-mut, GV272-miR-27a promoter-wild or GV272-miR-27a promoter-mut plasmids and miRNA-27a mimic, si-RARα or non-relative control RNA duplex (NC duplex; GenePharma) by using Lipofectamine™ 3000 in accordance with the manufacturer's procedure, respectively. pRL-TK (Promega, Madison, WI, USA) was transfected as a normalization control. Cells were collected at 24 h after transfection and luciferase activity was measured using a dual-luciferase reporter assay kit (Promega) and recorded by Chemiluminescence Meter (Promega).

### ChIP

Hep-2 cells were cross-linked with 1% formaldehyde for 10 min. ChIP assay was performed by using anti-RARα antibody (Abcam) and ChIP assay kit (Millipore, Billerica, MA, USA) according to the manufacturer's instructions. Anti-rabbit IgG antibody (Santa Cruz, CA, USA) was used as negative control. Bound DNA fragments were subjected to quantitative-PCR or PCR detection using the primer pair shown in Table 2. PCR products were separated by electrophoresis on 2% agarose gel.

### Statistical analysis

Data were subjected to statistical analysis by Graphpad 6.0 software and shown as mean ± standard deviation (SD) or mean ± standard error of the mean (SEM) based on at least three independent experiments. 2-tailed Student *t* test and one-way ANOVA were carried out. Symbols *, **, *** and ns represent *p* < 0.05, *p* < 0.01, *p* < 0.001 and no significance, respectively.

## SUPPLEMENTARY MATERIALS FIGURES AND TABLES


