# Supplementary material for: Transcriptional suppression of microRNA-27a contributes to laryngeal cancer differentiation via GSK-3β-involved Wnt/β-catenin pathway
Source: Oncotarget. 2017 Jan 20;8(9):14708–18. doi: 10.18632/oncotarget.14769 (PMC5362437; doi:10.18632/oncotarget.14769)
Supplement: Supplementary file 1 [file oncotarget-08-14708-s001.pdf]

Transcriptional suppression of microRNA-27a contributes to laryngeal cancer differentiation via GSK-3β-involved Wnt/β-catenin pathway

Supplementary Materials

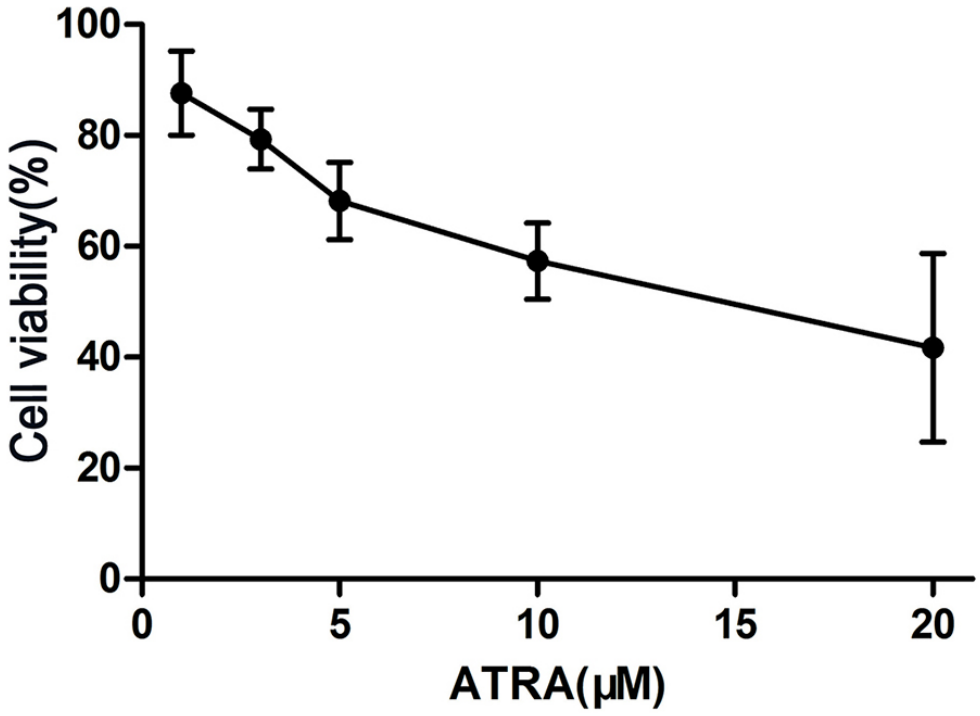

Supplementary Figure 1: Viabilities of laryngeal cancer cells treated by different concentrations of ATRA.

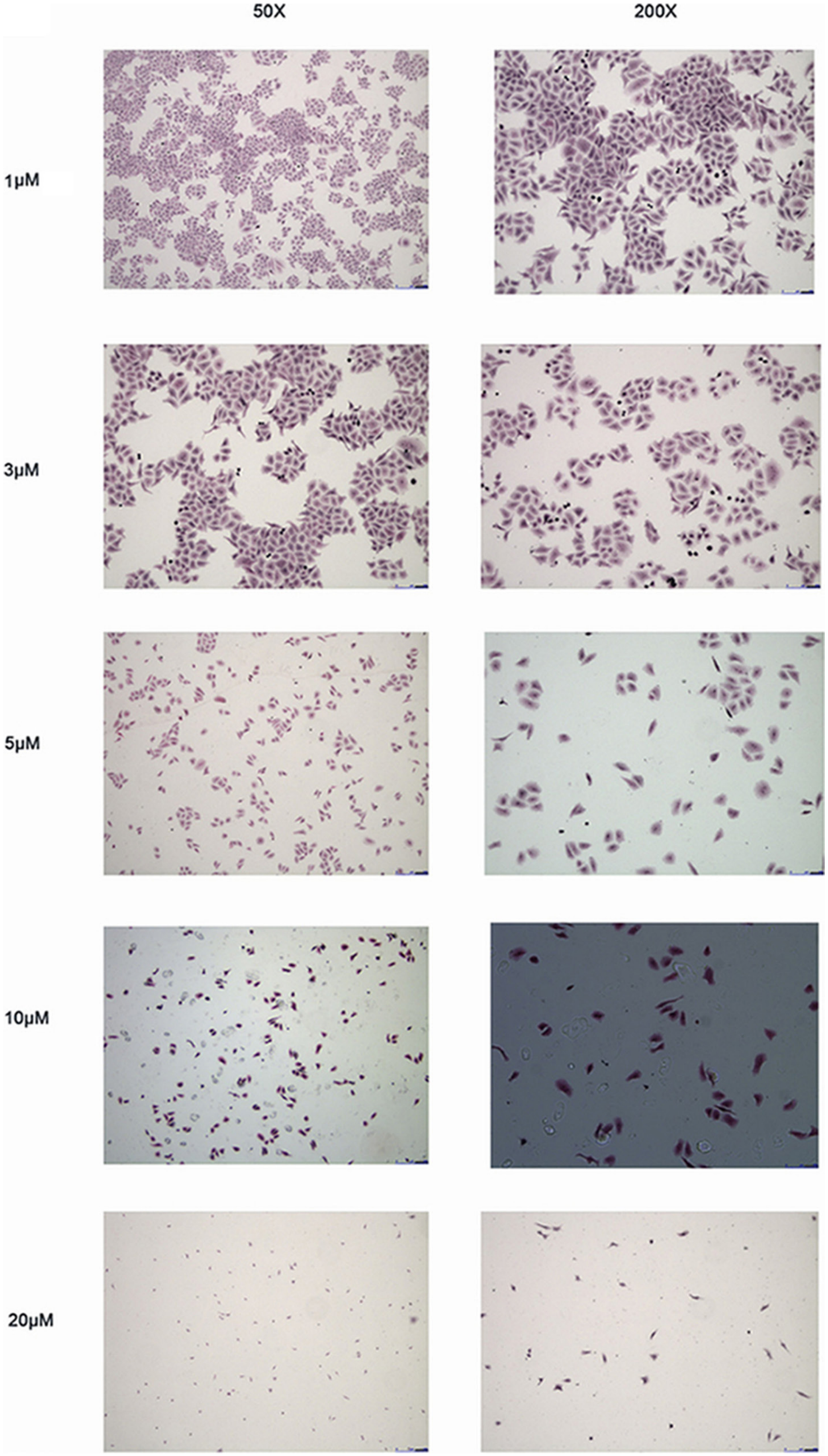

Supplementary Figure 2: Cell morphological alterations in laryngeal cancer cells treated by different concentrations of ATRA.
